# Risk Factors for the Occurrence and Progression of Posttraumatic Elbow Stiffness: A Case-Control Study of 688 Cases

**DOI:** 10.3389/fmed.2020.604056

**Published:** 2020-12-16

**Authors:** Yun Qian, Shiyang Yu, Yue Shi, Hengye Huang, Cunyi Fan

**Affiliations:** ^1^Department of Orthopedics, Shanghai Jiao Tong University Affiliated Sixth People's Hospital, Shanghai, China; ^2^Youth Science and Technology Innovation Studio, Shanghai Jiao Tong University School of Medicine, Shanghai, China; ^3^School of Public Health, Shanghai Jiao Tong University School of Medicine, Shanghai, China; ^4^Shanghai Sixth People's Hospital East Affiliated to Shanghai University of Medicine & Health Sciences, Shanghai, China

**Keywords:** elbow stiffness, risk factor, surgery, alcohol, trauma

## Abstract

**Background:** Elbow stiffness is a severe complication after trauma. Surgical or conservative treatments may be ineffective for restoring functional elbow motion. We aim to evaluate intrinsic and extrinsic factors for the occurrence and severity of elbow stiffness.

**Methods:** This retrospective case–control study included mild/moderate stiffness, severe stiffness, and non-stiffness groups between January 2011 and December 2017 at a single orthopedic center. Multivariable logistic regression analysis and subgroup analysis were used to evaluate age, gender, body mass index, muscle strength, fracture type and site, injury mechanism, immobilization time, elbow dysfunction time, multiple surgeries, nerve symptoms, physical therapy, smoking and alcohol abuse, and dominant hand of stiff elbow as potential risk factors for the occurrence and severity of elbow stiffness.

**Results:** There were 461 patients in the stiffness group and 227 patients in the non-stiffness group. The odds ratios (ORs) of the age, muscle strength, and injury mechanism were 0.960, 0.333, and 0.216 for the occurrence of elbow stiffness. In subgroup evaluation, increased cast immobilization time might be a risk factor for patients receiving conservative therapies (OR = 2.02; *p* = 0.014). In the evaluation on factors for progression of elbow stiffness, “multiple surgeries” might be a risk factor in surgical treatment by subgroup analysis (OR = 1.943; *p* = 0.026). Nevertheless, alcohol abuse might increase severity of elbow stiffness in conservatively treated patients (OR = 3.082; *p* = 0.025).

**Conclusion:** Increased cast immobilization time in the conservative therapy might be a risk factor for stiffness occurrence. Multiple surgeries might be risk factors for stiffness progression. Alcohol abuse potentially increased stiffness severity after conservative treatment.

## Introduction

Elbow motion loss results in considerable daily function and personal hygiene limitation. Elbow stiffness is characterized by restriction in flexion, extension, supination, or pronation (1–4). Trauma is the most common cause for elbow stiffness. Around 10–15% patients fail to recover from elbow injuries and experience motion limitation (5). The etiology mainly includes heterotopic ossification (HO), joint contracture, delay, or failure in bone repair and arthritic degeneration (6–10). The pathophysiological changes are inflammation and fibroblast proliferation in the elbow (11). Previous research reported different protocols on elbow stiffness treatment and interfering factors of improving the prognosis (12–15). Nevertheless, the treatment of elbow stiffness is difficult and the sequel is still not ideal. Many patients experience limited function improvement or even recurrent stiffness after operative or conservative treatment (16–18). Therefore, it is urgent and vital to investigate possible risk factors behind elbow stiffness. Some studies claimed extended elbow immobilization, severe fracture, and multiple surgeries within a short period of time as primary potential risk factors (19, 20). Comprehensive evaluation based on a large sample size is still lacking. In the present study, we discuss and analyze potential risk factors for the onset and progression of elbow stiffness after upper-extremity trauma for the first time. We hypothesize the involvement of some demographical and injury- and treatment-related factors in elbow stiffness.

## Methods

**Scheme 1:**
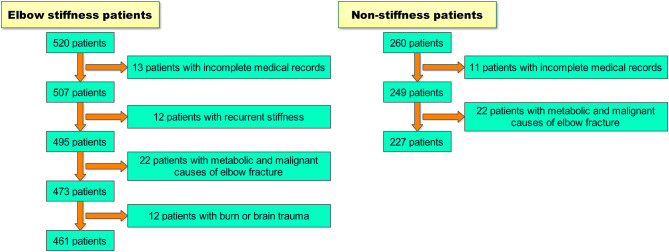
Patient selection of this study.

A retrospective case–control study was performed between January 2011 and December 2017 at Shanghai Jiao Tong University affiliated Sixth People's Hospital. Patients were reviewed who were diagnosed with elbow stiffness after upper-extremity trauma and had upper-extremity trauma but did not develop elbow stiffness. In the elbow stiffness group, the inclusion criteria were (1) skeletal mature and complete bone healing, (2) elbow range of motion (ROM) <100°, and (3) history of surgical intervention or conservative treatment for upper-extremity trauma. Exclusion criteria were (1) incomplete medical records, (2) recurrence of elbow stiffness, (3) metabolic and malignant causes of elbow fracture, and (4) burn or brain trauma. In the non-stiffness group, participants did not develop elbow stiffness after upper-extremity trauma. The inclusion criteria were (1) skeletal mature and complete bone healing, (2) elbow ROM ≥100°, and (3) history of surgical intervention or conservative treatment for upper-extremity trauma. Exclusion criteria were (1) incomplete medical records and (2) metabolic and malignant causes of elbow fracture. We reviewed all elbow stiffness patients from our medical history system between January 2011 and December 2017. Among all the 520 patients, we excluded 13 patients with incomplete medical records, 12 patients with recurrent stiffness, 22 patients with metabolic and malignant causes of elbow fracture, and 12 patients with burn or brain trauma. We also reviewed 260 non-stiffness patients by a 2:1 ratio using random sampling from our medical history system during the same period of time. We excluded 11 patients with incomplete medical records and 22 patients with metabolic and malignant causes of elbow fracture. Finally, we included 688 patients in this study, including 461 elbow stiffness patients (233 in the mild/moderate elbow stiffness group, 228 in the severe elbow stiffness group) and 227 non-stiffness patients (Scheme 1).

Mild/moderate elbow stiffness refers to elbow ROM of 50–100°, and severe elbow stiffness refers to elbow ROM of 0–50°. We included some intrinsic demographic factors and extrinsic injury-related factors for consideration from the patients' medical records in this study. Demographic information included age, sex, body mass index (BMI), smoking, alcohol abuse, and dominant hand. BMI was classified into underweight, normal-weight, overweight, and obese levels according to Chinese standard (21). Injury-related information included muscle strength, fracture type, fracture site, injury mechanism, immobilization time, elbow dysfunction time, rehabilitation exercise, nerve symptoms, and multiple surgeries (no fewer than 2 surgeries for the primary trauma and subsequent complications) history. Handgrip muscle strength was measured by a dynamometer around the time of treatment and categorized into normal muscle strength and muscle weakness. Fractures were categorized into open, closed, and combined (multiple fractures with both open and closed wounds) fractures. The injury mechanism involved low-energy trauma (falls from ground levels) and high-energy trauma (e.g., traffic accidents and falls from significant levels). The immobilization involved preoperative, postoperative, and conservative cast immobilization. Fracture sites included distal humerus fracture, radial head fracture, olecranon fracture, terrible triad, elbow dislocation, multiple fractures, and other peri-articular fractures that could fall into either category previously according to the AO classification system. Three independent senior surgeons for elbow surgery reviewed clinical information, including imaging and elbow ROM, and decided their classification of stiffness severity, fracture location, and types. Positive nerve symptoms referred to lasting numbness in upper extremities at least 3 months after an initial injury. We performed subgroup analysis based on treatment type (surgery or conservative therapy) because some variables only existed in either surgery or conservative treatment. Oral and informed consents were acquired from all participants in this study. The IRB approval was obtained from Shanghai Jiao Tong University affiliated Sixth People's Hospital East Campus (No. 2018-013).

### Statistical Analysis

The continuous variables with normal and skewed distribution were displayed by median (p25, p75), and the categorical variables were displayed by frequency (percent). Respectively, *T*-test or non-parametric rank-sum test was adopted to compare the difference between groups of continuous variables, and chi-square test (or Fisher's exact test, where applicable) or non-parametric rank-sum test was adopted to compare the difference between groups of qualitative data. Variables that showed significances between groups in univariate analysis were further evaluated in multivariate analysis using the logistic regression model. A *P-*value of <0.05 was considered as statistical significance. Statistical analyses were conducted by SPSS 22.0 (IBM, New York, USA).

## Results

### Factors in the Occurrence of Elbow Stiffness

In the demographic information, significant differences were found in age (*p* < 0.001), sex (*p* = 0.001), BMI (*p* = 0.009), smoking (*p* = 0.034), and alcohol abuse (*p* < 0.001) between elbow stiffness and non-stiffness groups (Table 1). In the injury-related information, there were significant differences in muscle strength (*p* < 0.001), injury mechanism (*p* < 0.001), and fracture site (*p* < 0.001) between the above two groups (Table 1). In the multiple logistic regression analysis, sex, BMI, smoking, or alcohol abuse showed insignificant differences between stiffness and non-stiffness groups. The odds ratios (ORs, 95% confidence interval, CI) of the age, muscle strength, and injury mechanism were 0.960 (0.947, 0.973), 0.333 (0.227, 0.488), and 0.216 (0.128, 0.365) (Table 2).

**Table 1:** Demographic and injury-related information in elbow stiffness group and non-stiffness group.

**Variables**	**Total (*N* = 688)**	**Non-stiffness group (*N* = 227)**	**Elbow stiffness group (*N* = 461)**	***P-*value**
Age (y)	35 (26, 47)	41 (29, 58)	33 (25, 43.5)	<0.001^**^
Sex				0.001^**^
Male	426 (61.9)	121 (53.3)	305 (66.2)	
Female	262 (38.1)	106 (46.7)	156 (33.8)	
Body mass index (kg/m^2^)				0.009^**^
Underweight	65 (9.4)	14 (6.2)	51 (11.1)	
Normal-weight	383 (55.8)	121 (53.3)	262 (56.8)	
Overweight	166 (24.1)	65 (28.6)	101 (21.9)	
Obese	74 (10.7)	27 (11.9)	47 (10.2)	
Muscle strength (N)	234 (34.1)	119 (52.7)	115 (25)	<0.001^**^
Fracture type				0.853
Closed	672 (97.7)	222 (97.8)	450 (97.6)	
Open	7 (1)	5 (2.2)	2 (0.4)	
Combined	9 (1.3)	0 (0)	9 (2)	
Low-energy trauma	95 (13.8)	64 (28.3)	31 (6.7)	<0.001^**^
Nerve symptom	184 (26.7)	57 (25.1)	127 (27.5)	0.497
Fracture site				<0.001^**^
Distal humerus fracture	184 (26.7)	62 (27.3)	122 (26.5)	
Radial head fracture	114 (16.6)	52 (22.9)	62 (13.4)	
Olecranon fracture	147 (21.4)	49 (21.6)	98 (21.2)	
Multiple fractures	151 (21.9)	37 (16.3)	114 (24.7)	
Terrible triad	41 (6.0)	15 (6.6)	26 (5.5)	
Elbow dislocation	47 (6.8)	12 (5.3)	35 (7.7)	
Other peri-articular fractures	4 (0.6)	0 (0)	4 (0.9)	
Smoking	189 (27.5)	74 (32.6)	115 (24.9)	0.034^*^
Alcohol abuse	366 (53.2)	210 (92.5)	156 (33.8)	<0.001^**^
Dominant hand	247 (51.4)	121 (53.3)	126 (27.3)	0.418

* *p < 0.05*;

** *p < 0.01*.

*y, years; N, newton*.

*Sex: male and female; body mass index (kg/m^2^): underweight, normal-weight, overweight, and obese. Fracture type: closed, open, and combined. Fracture site: distal humerus fracture, radial head fracture, olecranon fracture, multiple fractures, terrible triad, elbow dislocation, and other peri-articular fractures*.

**Table 2:** Multiple logistic regression analysis of significantly different variables between elbow stiffness and non-stiffness groups.

**Variables**	**Regression coefficient**	**OR (95%CI)**	***P-*value**
Age (y)	−0.041	0.960 (0.947, 0.973)	<0.001^**^
Muscle strength (N)	−1.099	0.333 (0.227, 0.488)	<0.001^**^
Low-energy trauma	−1.531	0.216 (0.128, 0.365)	0.001^**^

** *p < 0.01; OR, odds ratio; CI, confidence interval*.

*y, years; N, newton*.

In the subgroup analysis by surgery, some variables showed a statistically significant difference between elbow stiffness and non-stiffness groups, such as the age (*p* = 0.001), preoperative immobilization time (*p* < 0.001), postoperative immobilization time (*p* < 0.001), sex (*p* < 0.001), muscle strength (*p* < 0.001), and injury mechanism (*p* = 0.001) (Table 3). In the multiple logistic regression analysis, the onset of elbow stiffness might be inversely correlated with the age (OR = 0.95; 95% CI = 0.94, 0.97; *p* < 0.001), preoperative immobilization time (OR = 0.69; 95% CI = 0.57, 0.84; *p* < 0.001), postoperative immobilization time (OR = 0.90; 95% CI = 0.87, 0.94; *p* < 0.001), muscle strength (OR = 0.24; 95% CI = 0.15, 0.41; *p* < 0.001, and low-energy trauma (OR = 0.14; 95% CI = 0.07, 0.27; *p* < 0.001).

**Table 3:** Subgroup analysis and multiple logistic regression analysis on occurrence of elbow stiffness by treatment type (surgery).

**Subgroup analysis (surgery)**				**Multiple logistic regression analysis (surgery)**		
**Variables**	**Non-stiffness group (*N* = 211)**	**Elbow stiffness group (*N* = 358)**	***P-*value**	**Regression coefficient**	**OR (95%CI)**	***P-*value**
Age (y)	42 (29, 58)	32 (25, 43.3)	0.001^**^	−0.050	0.95 (0.94, 0.97)	<0.001^**^
Sex			<0.001^**^			
Male	112 (53.1)	248 (69.3)		–	–	–
Female	99 (46.9)	110 (30.7)		–	–	–
Muscle strength (N)	116 (55.2)	82 (23)	<0.001^**^	−1.409	0.24 (0.15, 0.41)	<0.001^**^
Preoperative immobilization time (w)	1 (1, 1)	0 (0, 0)	<0.001^**^	−0.374	0.69 (0.57, 0.84)	<0.001^**^
Postoperative immobilization time (w)	4 (0, 15)	0 (0, 4)	<0.001^**^	−0.101	0.90 (0.87, 0.94)	<0.001^**^
Low-energy trauma	62 (29.5)	27 (7.5)	0.001^**^	−1.964	0.14 (0.07, 0.27)	<0.001^**^
Smoking	70 (33.2)	91 (25.4)	<0.001^**^	–	–	–
Alcohol abuse	195 (92.4)	129 (36)	<0.001^**^	–	–	–

** *p < 0.01; OR, odds ratio; CI, confidence interval*.

*y, years; N, newton; w, weeks*.

“–” *indicated that variables showed no significance after logistic regression analysis*.

Conservative therapies were applied in 103 patients from the elbow stiffness group and 16 patients from the non-stiffness group. The subgroup analysis based on conservative treatment indicated that there were significant differences in variables, like the cast immobilization time (*p* = 0.003), nerve symptoms (*p* = 0.045), and alcohol abuse (*p* < 0.001) (Table 4). In the multiple logistic regression analysis, only immobilization time displayed a statistical difference between elbow stiffness and non-stiffness groups for conservative treatment (OR = 2.02; 95% CI = 1.15, 3.54; *p* = 0.014, Table 4).

**Table 4:** Subgroup analysis and multiple logistic regression analysis on occurrence of elbow stiffness by treatment type (conservative therapy).

**Subgroup analysis (conservative therapy)**				**Multiple logistic regression analysis (conservative therapy)**		
**Variables**	**Non-stiffness group (*N* = 16)**	**Elbow stiffness group (*N* = 103)**	***P-*value**	**Regression coefficient**	**OR (95%CI)**	***P-*value**
Cast immobilization time (w)	1 (0, 1)	4 (0, 6)	0.003^**^	0.703	2.02 (1.15, 3.54)	0.014^*^
Nerve symptom	0 (0)	27 (26.2)	0.045^*^	–	–	–
Alcohol abuse	15 (93.8)	27 (26.2)	<0.001^**^	–	–	–

* *p < 0.05*;

** *p < 0.01; OR, odds ratio; CI, confidence interval*.

*w, weeks*.

“–” *indicated that variables showed no significance after logistic regression analysis*.

### Factors in the Severity of Elbow Stiffness

In the demographic data, only “age” showed significant differences between mild/moderate and severe elbow stiffness groups (*p* = 0.001, Table 5). In the injury-related data, there were significant differences in muscle strength only (*p* = 0.031, Table 5). In the multiple logistic regression analysis, neither age nor muscle strength displayed any difference between mild/moderate and severe elbow stiffness groups.

**Table 5:** Demographic and injury-related information in severe and mild/moderate elbow stiffness groups.

**Variables**	**Total (*N* = 461)**	**Severe elbow stiffness group (*N* = 228)**	**Mild/moderate elbow stiffness group (*N* = 233)**	***P-*value**
Age (y)	33 (25, 43.5)	35 (27, 45)	31 (24, 41)	0.001^**^
Sex				0.340
Male	305 (66.2)	146 (64)	159 (68.2)	
Female	156 (33.8)	82 (36)	74 (31.8)	
Body mass index (kg/m^2^)				0.137
Underweight	50 (10.9)	25 (11.0)	25 (10.7)	
Normal-weight	264 (57.3)	120 (52.6)	144 (61.8)	
Overweight	101 (21.8)	60 (26.3)	41 (17.6)	
Obese	46 (10)	23 (10.1)	23 (9.9)	
Muscle strength (N)	115 (25)	67 (29.4)	48 (20.7)	0.031^*^
Fracture type				0.721
Closed	450 (97.6)	222 (97.4)	228 (97.9)	
Open	2 (0.4)	0 (0)	2 (0.9)	
Combined	9 (2)	6 (2.6)	3 (1.3)	
Low-energy trauma	31 (6.7)	13 (5.7)	18 (7.7)	0.386
Elbow dysfunction time (w)	14 (10, 21)	14 (10, 21)	14 (10.25, 21)	0.646
Nerve symptom	127 (27.5)	65 (28.5)	62 (26.6)	0.648
Rehabilitation exercise	184 (40.1)	88 (38.8)	96 (41.4)	0.568
Fracture site				0.155
Distal humerus fracture	122 (26.5)	61 (26.7)	61 (26.1)	
Radial head fracture	62 (13.4)	30 (13.2)	32 (13.7)	
Olecranon fracture	97 (21.0)	48 (21.1)	49 (21.2)	
Multiple fractures	115 (24.9)	62 (27.1)	53 (22.6)	
Terrible triad	25 (5.4)	11 (4.8)	14 (6.2)	
Elbow dislocation	36 (7.8)	12 (5.3)	24 (10.2)	
Other peri-articular fractures	4 (0.9)	4 (1.8)	0 (0)	
Smoking	115 (24.9)	59 (25.9)	56 (24)	0.648
Alcohol abuse	156 (33.8)	80 (35.1)	76 (32.6)	0.575
Dominant hand	125 (49.4)	76 (54.7)	49 (43)	0.064

* *p < 0.05*;

** *p < 0.01*.

*y, years; N, newton; w, weeks*.

*Sex: male and female. Body mass index (kg/m^2^): underweight, normal-weight, overweight, and obese. Fracture type: closed, open, and combined. Fracture site: distal humerus fracture, radial head fracture, olecranon fracture, multiple fractures, terrible triad, elbow dislocation, and other peri-articular fractures*.

In the subgroup analysis by surgery, the severity of elbow stiffness might be correlated with the age (*p* = 0.001) and multiple surgeries (*p* = 0.038) (Table 6). The age and multiple surgeries were evaluated in surgery groups by the multiple logistic regression analysis. “Multiple surgeries” might be associated with increasing severity of elbow stiffness (OR = 1.943; 95% CI = 1.081, 3.490; *p* = 0.026, Table 6). In the conservative therapies, there were significant differences in the age (*p* = 0.003), fracture site (*p* = 0.040), and alcohol abuse (*p* = 0.016) between mild/moderate and severe elbow stiffness groups (Table 7). Further analysis indicated that the age and alcohol abuse might be risk factors for increased severity of elbow stiffness (age: OR = 1.047; 95% CI = 1.011, 1.085; *p* = 0.011; alcohol abuse: OR = 3.082; 95% CI = 1.153, 8.237; *p* = 0.025, Table 7).

**Table 6:** Subgroup analysis and multiple logistic regression analysis on severity of elbow stiffness by treatment type (surgery).

**Subgroup analysis (surgery)**					**Multiple logistic regression analysis (surgery)**		
**Variables**	**Total (*N* = 358)**	**Severe elbow stiffness group (*N* = 176)**	**Mild/moderate elbow stiffness group (*N* = 182)**	***P-*value**	**Regression coefficient**	**OR (95%CI)**	***P-*value**
Age (y)	32 (25, 43)	34 (26, 44.8)	31 (25, 41)	0.001^**^	–	–	–
Multiple surgeries	58 (12.7)	36 (15.9)	22 (9.5)	0.038^*^	0.664	1.943 (1.081, 3.490)	0.026

* *p < 0.05*;

** *p < 0.01; OR, odds ratio; CI, confidence interval*.

*y, years*.

“–” *indicated that variables showed no significance after logistic regression analysis*.

**Table 7:** Subgroup analysis and multiple logistic regression analysis on severity of elbow stiffness by treatment type (conservative therapy).

**Subgroup analysis (conservative therapy)**					**Multiple logistic regression analysis (conservative therapy)**		
**Variables**	**Total (*N* = 101)**	**Severe elbow stiffness group (*N* = 51)**	**Mild/moderate elbow stiffness group (*N* = 50)**	***P-*value**	**Regression coefficient**	**OR (95%CI)**	***P-*value**
Age (y)	35 (25, 44)	39 (30, 49)	30 (21, 41)	0.003^**^	0.046	1.047 (1.011, 1.085)	0.011
Fracture site				0.040^*^			
Distal humerus fracture	19 (18.8)	11 (21.6)	8 (16.0)		–	–	–
Radial head fracture	21 (20.8)	13 (25.5)	8 (16.0)				
Olecranon fracture	19 (18.8)	5 (9.8)	14 (28.0)				
Multiple fractures	14 (13.9)	6 (11.8)	8 (16.0)				
Terrible triad	4 (4.0)	3 (5.9)	1 (2.0)				
Elbow dislocation	20 (19.8)	9 (17.6)	11 (22.0)				
Other peri-articular fractures	4 (4.0)	4 (7.8)	0 (0)				
Alcohol abuse	27 (26.3)	19 (36.5)	8 (15.7)	0.016	1.126	3.082 (1.153, 8.237)	0.025

* *p < 0.05*;

** *p < 0.01; OR, odds ratio; CI, confidence interval*.

*y, years*.

*Fracture site: distal humerus fracture, radial head fracture, olecranon fracture, multiple fractures, terrible triad, elbow dislocation, and other peri-articular fractures*.

“–” *indicated that variables showed no significance after logistic regression analysis*.

## Discussion

Elbow injuries are common in trauma. At present, the treatment is ineffective and therefore it is urgent and important to investigate the possible risk factors. In this study, elbow stiffness patients due to traumatic elbow injuries were included instead of other origins. We evaluated risk factors in the occurrence of traumatic elbow stiffness. Younger age, lower muscle strength, and high-energy trauma were associated with the occurrence of elbow stiffness. These variables were validated further in subgroup analysis.

Previously, there were few studies on the potential influence of age in the onset of elbow stiffness. In this study, older age was found to potentially decrease risks for elbow stiffness. According to our observation and literature, young patients tend to develop severe elbow contracture and motion limitation. Their skeletomuscular system is developing rapidly. It may stimulate excretion of different growth factors and contribute to bone regeneration and irregular HO formation (22). The finding was also confirmed in another recent study. Carlock et al. reported that patients with elbow contracture were younger than the non-contracture ones (47.1 vs. 54.9 years, *P* = 0.004) (23).

Severe stiffness patients suffered from high-energy injuries and were treated by multiple-hardware fixation. They might have a bigger chance of developing stiff elbows due to prolonged postoperative immobilization time (24). There were some debates on the immobilization time. Some literature recommended early postoperative activity because it was good for maintaining elbow motion and preventing stiffness, but protocols varied in different studies. Taylor et al. reported that early mobilization at 3 days after reduction of elbow dislocation increased elbow motion compared by cast immobilization. However, their results were statistically insignificant (25). Increased immobilization time in surgery groups appeared to be a beneficial factor, which was contrasted with its role in conservative therapy. Temporary cast immobilization before surgery was good for anatomical reduction during surgery. It also avoided additional damages to neighboring tissues (26). The connective tissues, such as peripheral nerves, tendons, and ligaments were usually released and repaired intra-operatively. Suitable postoperative immobilization is beneficial for restoring the local structure and regenerating impaired tissues. However, few patients received surgeries within 24 h after injury in this study. This fact might cause bias on the role of immobilization time in the surgical treatment. An appropriate length of immobilization time should be investigated in the future.

For conservative therapies, increased immobilization might be a risk factor for elbow stiffness occurrence. For instance, for each additional week, the risk of elbow stiffness increased by 1.02-fold. Monument et al. believed that prolonged cast immobilization was harmful to elbow motion because it might stimulate capsule contracture and fibrosis and cause structural deformation within the periarticular areas (27). Muscle strength was positively associated with lower occurrence of elbow stiffness. This point was in agreement with some previous literature (28, 29). Lengthy immobilization due to severe trauma, unwillingness, or pain caused muscle atrophy and capsular contracture (30).

We preliminarily found out that the age and muscle strength were correlated with increased severity of elbow stiffness in univariate analysis. However, they were later excluded after multiple logistic regression analysis.

Then, patients were categorized by treatment type so that surgical intervention could be evaluated as a primary factor in deterioration of elbow stiffness. In this study, “multiple surgeries” might be a risk factor in surgical treatment by subgroup analysis (OR = 1.943; *p* = 0.026). In patients receiving surgeries, it was found to be the only prominent risk factor that might cause severe elbow stiffness. The reasons for multiple surgeries mainly include dislocation, material failure, and change from external fixator to plate. Compared with those who received a single surgery, patients who received multiple surgeries had a 1.943-fold higher risk of developing severe elbow stiffness. Jupiter et al. found that multiple surgeries within 1 to 2 weeks after injury were more likely to lead to HO formation and elbow contracture (31). Modi et al. reported similar findings in the elbow dislocation treatment. They noticed that multiple reduction attempts increased risks of subsequent elbow arthrolysis (32). Wiggers et al. found that multiple surgeries within the first 4 weeks after elbow trauma were a prominent risk factor for HO formation in elbow motion limitation (33). Zheng et al. claimed that they did not find any differences in additional surgeries among mild, moderate and severe elbow stiffness, which might be due to a small sample size (34). Nevertheless, a previous research claimed that patients experienced capsule adhesion and elbow contracture after multiple surgeries, possibly due to local inflammation and elbow joint fibrosis (35).

The age and alcohol abuse were both associated with increased severity of elbow stiffness in patients who received conservative therapies. Alcohol abuse was also identified as a potential risk factor in severe elbow stiffness among conservative therapy groups. Alcohol abuse might increase severity of elbow stiffness in conservatively treated patients (OR = 3.082; *p* = 0.025). Alcohol-addicted patients bore a 3.082-fold higher risk of developing severe elbow stiffness than non-abuse patients. Alcohol addiction was harmful to bone development because it represented a major reason for bone loss (36). It also resulted in osteoarthritis after trauma and joint structure deformation which potentially worsened elbow stiffness (37).

As to the other risk factors, each additional age put a 1.047-fold higher risk on patients receiving conservative therapies. This was contradictory to the results in multiple logistic regression analysis for the onset of elbow stiffness. For elderly patients, they had a higher likeliness for developing multiple systemic complications that affected immune and hematopoietic systems. Immune diseases and compromised nutritional status might partially increase the severity of elbow stiffness because of the potential effects on elbow structure destruction (38).

The sex, BMI, smoking, and dominant hands did not show any relevance with occurrence or severity of elbow stiffness. Various fracture sites may have different effects on elbow stiffness. In this study, fracture sites showed no relationship with occurrence or progression of this issue. Numbness of upper extremity displayed no differences in any group possibly due to nerve compression beyond elbow contracture in both flexion and extension. Cai et al. reported that the ulnar neuritis was related with elbow flexion instead of extension limitation (39). The nerve symptoms might not be directly correlated with the severity of elbow stiffness. The rehabilitation exercise should improve elbow function recovery. However, many patients developed severe elbow stiffness after violent and incorrect elbow practices (40). The physicians should avoid stressing the healing bones and ligaments over a specific limit and cater to the individualized demand.

There are a few limitations of this study. The main limitation is that this study is based on retrospective evaluation of a database and only a correlation can be identified. In addition, we excluded some patients who had incomplete medical records. The subgroup analysis was exploratory to identify potential confounders in the regression analysis. Thirdly, there is a lack of information on whether the stiffness is induced by structural impingement or capsule fibrosis. The degree of arthrosis as an influencing factor, such as HO occurrence, is also not described. Several other factors that cannot be assessed with this study may be relevant, such as osseous congruency of the elbow, coping strategies, and compliance with physiotherapy treatment. Finally, risk factors such as alcohol, age, immobilization, and multiple surgical procedures are also identified as risk factors without defining the cutoff. We would like to perform prospective randomized controlled trials based on these issues in the future.

## Conclusions

The study demonstrates that the increased immobilization time in conservatively treated patients may be risk factors for the occurrence of elbow stiffness. For the severity of elbow stiffness, “multiple surgeries” may increase risks in the surgical treatment between mild/moderate and severe stiffness groups. Alcohol abuse may increase risks for stiffness progression in patients after conservative therapy. The findings are promising for prevention of elbow stiffness.

## Data Availability Statement

The original contributions presented in the study are included in the article/supplementary materials, further inquiries can be directed to the corresponding author/s.

## Ethics Statement

The studies involving human participants were reviewed and approved by Shanghai Jiao Tong University affiliated Sixth People's Hospital East Campus. The patients/participants provided their written informed consent to participate in this study.

## Author Contributions

CF and YQ conceived the initial idea and study design. YS and HH participated in data extraction and statistical analysis. YQ drafted the manuscript. YQ, SY, and CF revised the manuscript. All authors approved the final manuscript.

## Conflict of Interest

The authors declare that the research was conducted in the absence of any commercial or financial relationships that could be construed as a potential conflict of interest.
